# National and Subnational Population-Based Incidence of Cancer in Thailand: Assessing Cancers with the Highest Burdens

**DOI:** 10.3390/cancers9080108

**Published:** 2017-08-17

**Authors:** Shama Virani, Surichai Bilheem, Wasan Chansaard, Imjai Chitapanarux, Karnchana Daoprasert, Somsak Khuanchana, Atit Leklob, Donsuk Pongnikorn, Laura S. Rozek, Surattaya Siriarechakul, Krittika Suwanrungruang, Sukit Tassanasunthornwong, Patravoot Vatanasapt, Hutcha Sriplung

**Affiliations:** 1 Epidemiology Unit, Faculty of Medicine, Prince of Songkla University, Hat Yai 90110, Thailand; shamav@umich.edu (S.V.); surichai.bil@gmail.com (S.B.); 2 Environmental Health Sciences, School of Public Health, University of Michigan, Ann Arbor, MI 48109, USA; rozekl@umich.edu; 3 Cancer Registry Unit, Surat Thani Cancer Hospital, Surath Thani 84100, Thailand; wasan1013@gmail.com (W.C); sukitsi91@gmail.com (S.T.); 4 Chiang Mai Cancer Registry, Maharaj Nakorn Chiang Mai Hospital, Faculty of Medicine, Chiang Mai University, Chiang Mai 50200, Thailand; imjai@hotmail.com; 5 Cancer Registry Unit, Lampang Cancer Hospital, Lampang 52000, Thailand; kdaoprasert@gmail.com (K.D.); donsukp@hotmail.com (D.P.); 6 Cancer Unit, Lopburi Cancer Center, Lopburi 15000, Thailand; s098030971@yahoo.co.th (S.K.); s098030971@yahoo.co.th (A.L.); catsurattaya@hotmail.com (S.S.); 7 Cancer Unit, Srinagarind Hospital, Faculty of Medicine, Khon Kaen University, Khon Kaen 40002, Thailand; ksuwanrungruang@gmail.com (K.S.); patvat@kku.ac.th (P.V.)

**Keywords:** Thailand, incidence, trends, projections, health policy

## Abstract

In Thailand, five cancer types—breast, cervical, colorectal, liver and lung cancer—contribute to over half of the cancer burden. The magnitude of these cancers must be quantified over time to assess previous health policies and highlight future trajectories for targeted prevention efforts. We provide a comprehensive assessment of these five cancers nationally and subnationally, with trend analysis, projections, and number of cases expected for the year 2025 using cancer registry data. We found that breast (average annual percent change (AAPC): 3.1%) and colorectal cancer (female AAPC: 3.3%, male AAPC: 4.1%) are increasing while cervical cancer (AAPC: −4.4%) is decreasing nationwide. However, liver and lung cancers exhibit disproportionately higher burdens in the northeast and north regions, respectively. Lung cancer increased significantly in northeastern and southern women, despite low smoking rates. Liver cancers are expected to increase in the northern males and females. Liver cancer increased in the south, despite the absence of the liver fluke, a known factor, in this region. Our findings are presented in the context of health policy, population dynamics and serve to provide evidence for future prevention strategies. Our subnational estimates provide a basis for understanding variations in region-specific risk factor profiles that contribute to incidence trends over time.

## 1. Introduction

**Figure 1:**
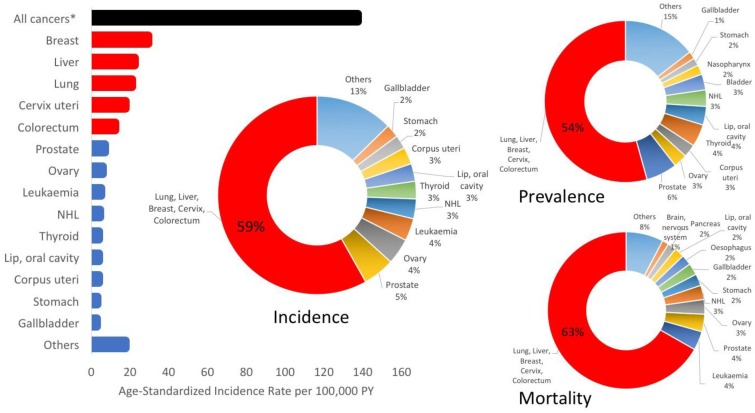
Breast, cervix, colorectal, liver and lung cancers combined had the highest age-standardized rates in 2012 and accounted for more than half of the incidence, prevalence and mortality in Thailand. *excluding non-melanoma skin cancer.

Approximately 60% of the cancer burden in Thailand is due to five types of cancers: breast, cervix, colorectal, liver and lung cancers [1] (Figure 1). Excluding melanoma, these five cancers accounted for 59.2% of incidence, 63.1% of mortality and 54.3% of 5-year prevalence in 2012 [1]. While these national estimates are useful in highlighting important cancers on a large scale, there is clear variability in the incidence of these cancers across the north, northeast, central and south regions [2,3,4,5,6,7]. Effective management of these cancers and attenuation of the future burden requires subnational estimates to determine differences in incidence by region.

There are many factors that differ by region and may contribute to region-specific cancer incidence profiles. Each region is culturally unique and this translates into diverse lifestyles. Regions can be generally characterized by culture, geography and diet. In the north, the culture is considered “lanna”, a unique mix of Siamese, Laotian and Burmese culture and traditions. The geography is mountainous and cool, offering suitable climate for agriculture of coffee, rice, and temperate-zone fruits and plants. The cool climate serves as the rationale for the consumption of fatty dishes in the diet, which is influenced by various minority groups including the Tai Yai, Haw Chinese, Tai Lue, and Burmese. The diet consists of boiled vegetables, herbs and roots as found naturally in the region, high consumption of glutinous rice and meat (mostly pork), in dishes served with mostly neutral flavors [8,9]. In the northeast region, culture is a mix of Siamese, Laotian and Khmer. This region has a primarily arid climate although rice cultivation is possible in some areas. Due to land infertility, diet in this region consists of high consumption of glutinous rice, raw/fermented river fish, insects and raw/fermented meats with very spicy, salty and sour flavors [8,9]. The culture in the central region is influenced by many Asian/European countries, due to its long-standing role as a hub for foreign trade. This region is situated in a fertile basin conducive to wet-rice agriculture, fruit farming and fishing industries. Diet consists of mainly steamed rice, vegetable and meat curries with coconut milk, distinctly combining sweet, salty, sour and spicy flavors [8,9]. Finally, the culture of the south contains contributions from the Malays and Chinese. This region is on a peninsula linked to the Malay Peninsula, with lots of heavy rain resulting in lush lands. Main industries include fishing, and palm, coconut and rubber tree plantations. Southern Indian, Malaysian and Javanese influences comprise a diet with heavy use of turmeric and capsicum, high consumption of seafood and with spicy and sour flavors. Pork consumption is lower compared to other regions due to a Muslims subpopulation that comprises 30% of the southern population and is the second most prevalent religion in Thailand after Buddhism [8,9].

Thailand became an upper middle income country in 2012, and rapid changes in the economy led to changes in lifestyles and behaviors, particularly with regard to those with higher incomes [10,11]. The shift in risk factor profiles likely plays a role in changing trends of cancer trends. However, aside from the role of environmental and lifestyle risk factors, incidence is also shaped by healthcare infrastructure and its ability to capture and diagnose cases when they occur. Since the 1970s, the Thai government offered insurance schemes to offset or support costs of healthcare for various population sectors. However, in 2001, approximately 30% of the population was still uninsured. In 2002, the Universal Health Coverage (UHC) system was introduced. Civil servants and their dependents remained under the Civil Servant Medical Benefit Scheme (CSBMS), formal sector employees continued to have their health-care costs paid for by the contributory Social Security Scheme (SSS) and previously uninsured people and those covered under the Medical Welfare Scheme and the Voluntary Health Card Scheme were covered under the Universal Coverage Scheme (UCS) [12,13]. Upon implementation, 97% of the population was covered under the UHC system [12,13,14,15]. The success of UHC was largely dependent upon investment in healthcare infrastructure and expansion of the healthcare workforce since the 1970s. By the time UHC was established, infrastructure existed at every level of the administrative system, from provincial hospitals to community healthcare centers [13]. This infrastructure improved access to health services, equity of service utilization and prevented medical impoverishment. The success of UHC was evident in Thailand’s achievement of the Millennium Development Goals (MDG), which were attained well before 2015. By the early 2000s, the MDG target of halving the population living in poverty was attained. Immunization coverage of diphtheria, tetanus and pertussis increased to 98%, prevalence of modern contraception increased to 73%, excess child mortality decreased and rich/poor and urban/rural gaps in maternal/child health services were reduced [16,17,18,19,20].

**Figure 2:**
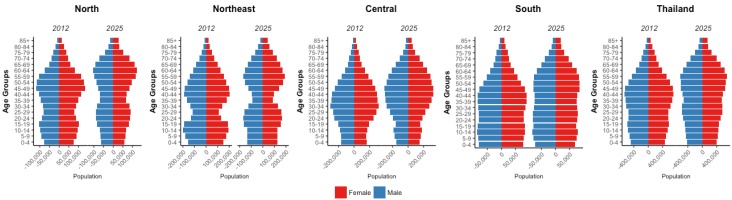
Population distribution for each region and nationally in 2012 and the expected population distribution in 2025.

According to the Ministry of Public Health, Thailand is undergoing an epidemiological transition. The prevalence rates of communicable diseases, which used to be significant, have declined with the exception of the reemergence of HIV and tuberculosis. Other major and rising causes of death include non-communicable diseases (NCDs) and accidents [10]. This increase in NCDs could be attributed to increased life expectancy, which increased by four years between 2000 and 2012 to 72 years for males and 78 years for females, and increases in aging populations (Figure 2) [21]. Non-communicable diseases account for 71% of deaths in Thailand, of which cancer accounts for the second largest proportion (17%) [22]. It is clear that Thailand’s epidemiologic transition will lead to an increase in cancer, making it crucial to provide estimates and projections to offer evidence that can be used to attenuate this burden in the future. The relatively recent introduction of UHC makes it difficult to assess mortality, as it will likely take several more years to see these effects. Nevertheless, we can quantify and assess cancer incidence with regard to UHC as improved access allows for better cancer detection, better estimates off which to base projections, and gives further weight to the fact that trends are shaped by risk factor profiles. Therefore, the aims of this research are focused on assessment of national and subnational cancer incidence trends over time of the top five cancers in Thailand and provide estimation for future burdens. Further, national and regional trends will be considered in the context of health policy in Thailand.

## 2. Results

### 2.1. National Trends, Projected Rates and Numbers

**Figure 3:**
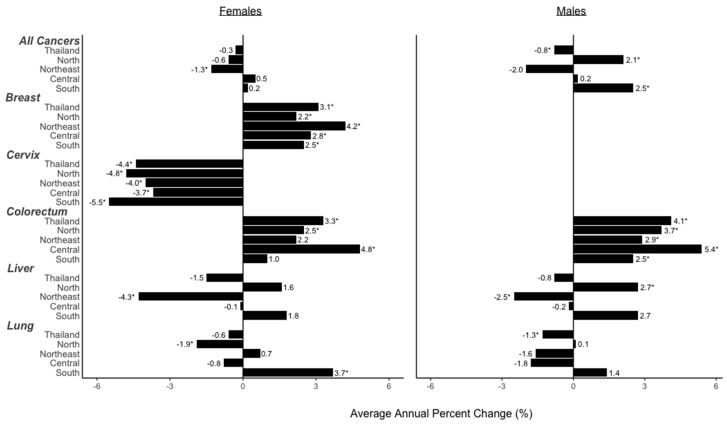
National and subnational average annual percent changes (AAPC) of each cancer from 2000 to 2012 for females and males. Significant AAPC are denoted with an asterisk (*). Significance was achieved at α = 0.05.

National cancer incidence rates were available from 2000 to 2012. Average annual percent changes (AAPCs) were calculated to provide a summary measure of change over the entire the time period. In females, incidence of all cancers combined remained constant over this time likely due to the combination of the increase in breast (AAPC: 3.1, 95% CI: 2.1, 4.1) and colorectal (AAPC: 3.3, 95% CI: 1.9, 4.8) cancers with the decrease in cervical cancer (AAPC: −4.4, 95% CI: −6.2, −2.6) (Figure 3). Projections were based on observed data using age-period-cohort models (Methods, Section 4.4). Breast cancer had the highest incidence of female cancers in 2012 and is expected to remain the highest in 2025. Colorectal cancer is expected to increase in incidence at the same rate as breast cancer from 2012 to 2025 and by 2025, both cancers combined will account for 63% of female cancer cases. Cervical cancer had the largest projected decline in incidence and is expected to account for only 7% of female cancers in 2025 (Table 1).

In males, incidence of all cancers declined from 2000 to 2012 (AAPC: −0.8, 95% CI: −1.5, −0.1), likely due to the significant decrease in lung cancer (AAPC: −1.3, 95% CI: −1.9, −0.7) (Figure 3). Liver cancer had the highest incidence of male cancers in 2012 and is projected to remain the highest in 2025. In 2012, liver and lung cancers contributed 80% of male cancer cases. While incidences of these are expected to decrease, colorectal cancer incidence is projected to increase, accounting for 29% of male cancer cases, while liver and lung cancers account for 71% of cases in 2025 (Table 1).

### 2.2. Regional Trends, Projected Rates and Numbers

**Figure 4:**
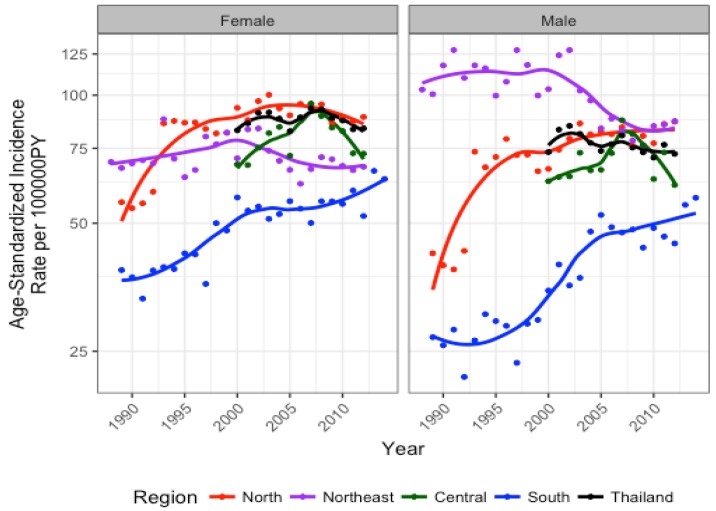
National and subnational estimates for incidence of the top five cancers combined for females and males.

Trend analysis showed that cancer incidence for the five cancers combined varied across regions and across time. Overall, cancer incidence has been highest in the north for females and northeast for males, although by 2012, male age-standardized incidence rates (ASRs) were similar in the north and northeast. Conversely, the south exhibited the lowest rates compared to all other regions for both sexes, albeit with an increasing trend (Figure 4; Table 1). From projections, it is expected that while female ASR for all cancers decrease in the north, they remain highest in this region in 2025. Female ASRs for all cancers in the south are expected to increase, but remain the lowest compared to other regions. Despite intermediate ASRs for all cancers in the central and northeastern regions in 2025, these regions are expected to contribute to the largest proportions of national female cancer cases (Table 1). In males, ASRs for all cancers are expected to become highest in the northern region and the lowest in the northeast. Although ASRs for all cancers increase in the central region and more so in the south, these regions are expected to reach intermediate ASRs compared to other regions. Nevertheless, the central and northeastern regions are projected to contribute the largest proportion of male cancer cases in Thailand in 2025.

**Figure 5:**
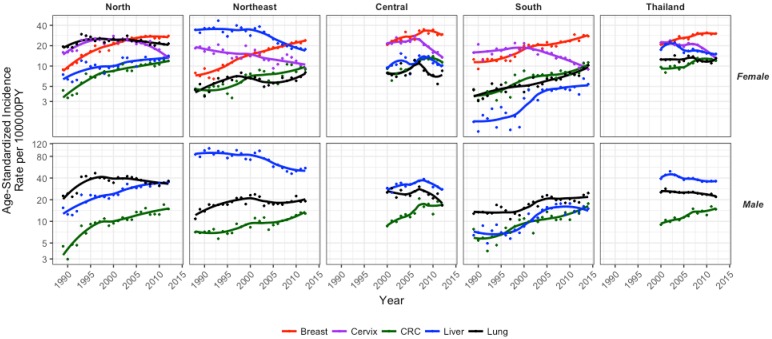
Incidence trends for each cancer by region and sex. Note: Different *Y*-axis range for each sex.

For all female cancers combined, the northeast was the only region that exhibited a significant decrease in average incidence per year from 2000 to 2012 (AAPC: −1.3, 95% CI: −2.4, −0.1). Across site-specific female cancers, breast cancer increased and cervical cancer decreased in all regions from 2000 to 2012. Colorectal cancer increased in the north (AAPC: 2.5, 95% CI: 1.2, 3.8) and central (AAPC: 4.8, 95% CI: 2.1, 7.5) regions. Liver cancer decreased in the northeast (AAPC: −4.3, 95% CI: −6.0, −2.5) and lung cancer increased in the south (AAPC: 3.7, 95% CI: 1.9, 5.6) (Figure 3, Figure 4, and Figure 5).

For all male cancers combined, the north and south regions experienced a significant increase in average incidence per year from 2000 to 2012 (AAPC: 2.1, 95% CI: 0.1, 4.2; AAPC: 2.5, 95% CI: 0.1, 4.9, respectively). For site-specific cancers, colorectal cancer incidence increased for all regions. Liver cancer increased in the north (AAPC: 2.7, 95% CI: 1.5, 4.0) and decreased in the northeast (AAPC: −2.5, 95% CI: −3.4, −1.5) (Figure 3, Figure 4, and Figure 5).

**Figure 6:**
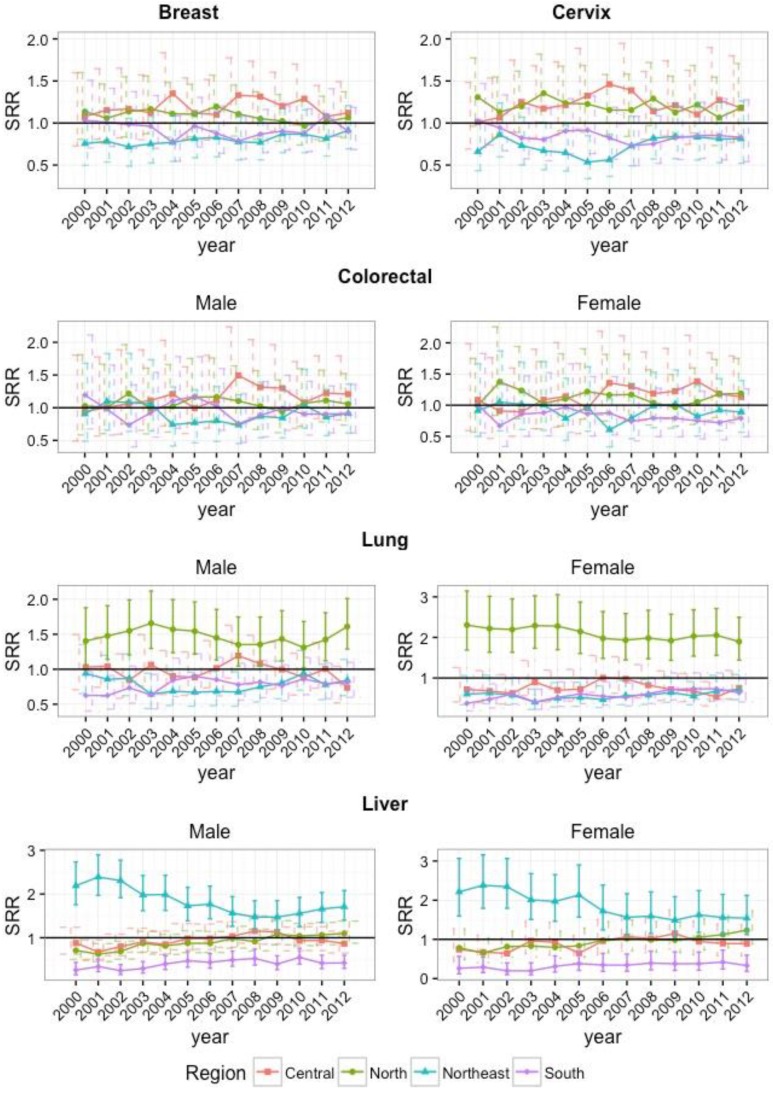
Standardized rate ratios (SRRs) and their 95% confidence intervals comparing incidence of each region to national estimates (SRR = 1) from 2000 to 2012. Solid lines for confidence intervals indicate significant SRRs and were only highlighted if SRRs were significant for all years. Significance was achieved at α = 0.05.

Standardized rate ratios (SRRs) of incidence were used to represent the relative risk of cancers by comparing each region to the national incidence (Figure 6). From 2000 to 2012, there were significantly higher relative risks of lung cancer in the north for both males and females. Similarly, higher relative risks were observed for liver cancer in the northeast, whereas lower relative risks were seen in the south, for both males and females. For all other cancers, SRRs for each region are not significantly different from national ASRs.

#### 2.2.1. North

Incidence trends were assessed in the north from 1989 to 2012 using segment analysis (Methods, Section 4.4). In females, breast cancer incidence increased significantly until 1994 and then remained stable. Female incidence of colorectal and liver cancers increased significantly from 1989 to 2012. Rates of female lung cancer decreased annually since 1993 while cervical cancer incidence largely decreased in last 5 years. In 2012, ASRs were highest for breast and lung cancers and these cases made up the over half of all female cancers cases in the north (55%). Projections indicated that breast cancer would have the highest ASR and proportion of cases of female cancers in this region in 2025, while cervical cancer will have the lowest. Projected ASRs for female colorectal cancers exhibit the largest percent increase in ASR from 2012 to 2025 compared to other cancers (14%), liver cancer ASRs are expected to remain stable and lung cancers ASRs are expected to decrease by 28%. Nevertheless, these cancers are expected to contribute in similar proportions (~20%) to the number of female cases in this region in 2025.

**Figure 7:**
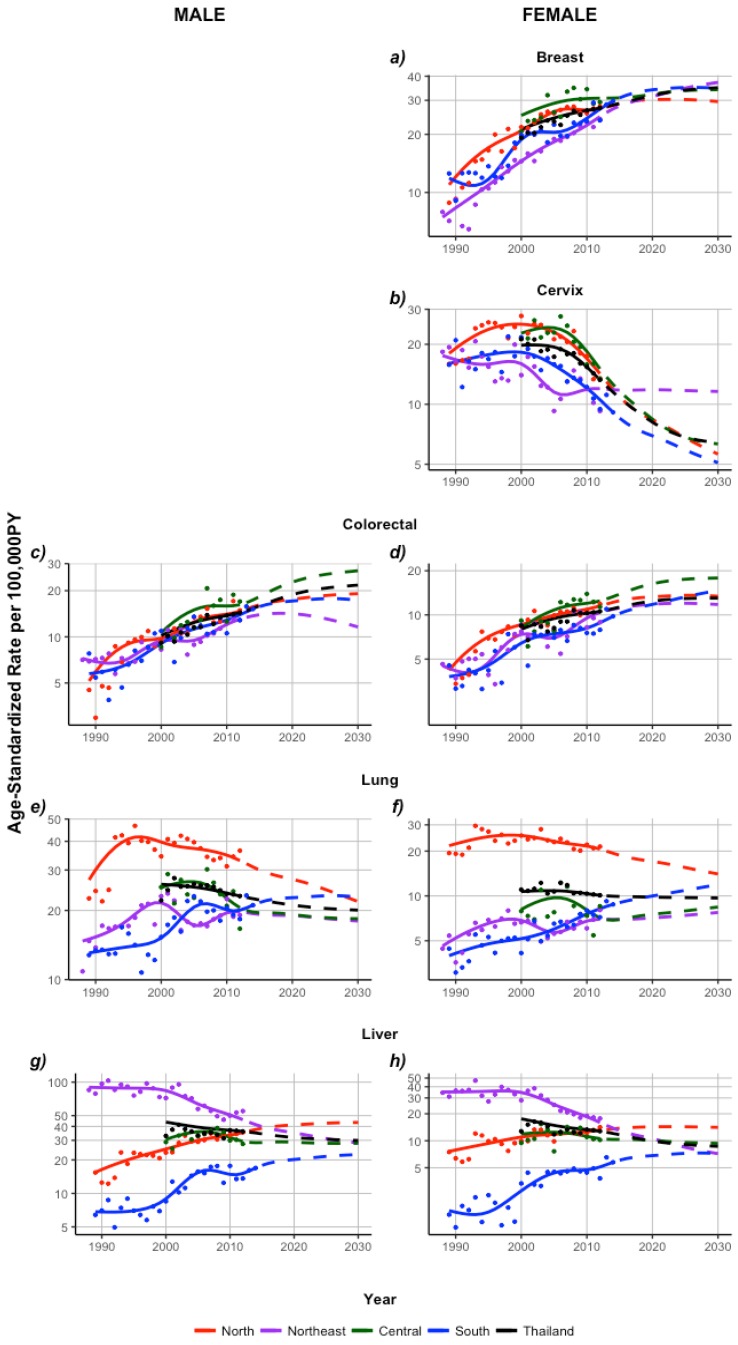
Projections of cancer trends for males and females by cancer

In males, colorectal and liver cancers increased from 1989 to 2012. Lung cancer incidence increased significantly until 1994 and then decreased by 1.3% annually. In spite of this, liver and lung cancer cases made up 80% of male cancer cases in this region in 2012. Projections indicate that while incidence of colorectal and liver cancers will increase by 20–25%, liver cancer will have the highest ASR and account for approximately half of male cancer cases in this region by 2025 (Figure 5 and Figure 7; Table 2).

#### 2.2.2. Northeast

Age-standardized rates in the northeast were quantified from 1988 to 2012. Female breast cancer increased throughout this period, accounting for the highest ASR of 24 cases per 100,000 person-years and largest proportion of cases of female cancers in this region by 2012. Colorectal cancer incidence increased by 3.5% annually while cervical cancer decreased by 2.3% annually, resulting in similar burdens in 2012. In the last decade, liver cancer decreased by 7.4% per year and lung cancer incidence increased by 4.5% per year.

In 2012, liver cancer was the second largest contributor to female cancer cases while cervical, colorectal and lung cancers accounted for similar proportions of cases. Projections illustrated incidence of breast, cervical and colorectal cancers are expected to increase while rates for liver and lung cancers are expected to decrease.

By 2025, it is expected that breast cancer cases continues to make up the largest proportion of female cancer cases followed by colorectal cancer cases. Lung cancer cases are expected to contribute the least to the total number of female cancer cases in this region in 2025.

Incidence of male liver cancer decreased by 8.4% from 2002 to 2008, while incidence of colorectal cancer increased annually by 2.8%. Despite these trends, these cancers made up the largest and smallest proportions of cases in 2012, respectively. Lung cancer increased until 2001 then remained stable. Colorectal and lung cancer incidence is projected to remain stable while liver cancer incidence is expected to decrease further by approximately 40%. Again, despite this decrease, almost half of all male cancer cases are expected to be attributable to liver cancer cases, followed by lung cancer cases in 2025 (Figure 5 and Figure 7; Table 2).

#### 2.2.3. Central

Central region incidence trends span the period from 2000 to 2012. Breast and colorectal cancer in females increased by 2.8% and 4.8% annually in this region from 2000 to 2012, respectively, while liver and lung cancer incidence remained relatively stable. Cervical cancer incidence decreased in recent years by 10% annually. Breast cancer accounted for the largest proportion of female cancer cases in 2012 whereas lung cancer cases contributed the smallest proportion of cases. Projections exhibit increasing incidence trends for breast and colorectal cancer, which are expected to continue to account for the largest and second largest proportions of female cancer cases, respectively, by 2025. In particular, the proportion of colorectal cancer cases is expected increase by 157% in 2025. Cervical cancer incidence will continue to decrease and contribute the smallest number of cases in 2025. 

Incidence of male colorectal cancer increased by 5.5%, while lung cancer decreased by 1.8% annually. Liver cancer incidence increased early and then decreased starting in 2008. In 2012, liver cancer accounted for almost half of male cancer cases in this region, while colorectal and lung cancer cases contributed to about a quarter of male cancer cases. Projections indicate that while colorectal cancer incidence will increase by 50% and liver cancer incidence will remain relatively stable, they will account for similar proportions of male cancer cases in 2025 (Figure 5 and Figure 7; Table 2).

#### 2.2.4. South

Incidence rates were assessed in the south from 1989 to 2014. In females, breast, colorectal, liver and lung cancer incidence increased by 2.1%, 4.0%, 5.5% and 4.7% annually, throughout this observed period. Cervical cancer incidence decreased by 5% annually starting in 2000. By 2012, breast cancer cases contributed the highest to female cancer cases, while liver cancer cases contributed the lowest. Projections indicate that by 2025, ASRs for breast, colorectal, liver and lung cancers will increase by approximately 50% or greater while cervical cancer will continue to decrease. It is expected that in 2025, breast cancer cases will continue to contribute the most to female cancer cases and cervical cancer will contribute the least.

In males, colorectal cancer incidence increased by 4.2% annually. Liver and lung cancer incidence showed significant increases in the early 2000s before stabilizing. Lung cancer cases made up about 40% of male cancer cases in this region while colorectal and liver cancer cases contributed approximately 30% each to male cancer cases. Projections estimate incidence trends for all three cancers to continue to increase with lung cancer exhibiting the highest ASR of 23.2. In 2025, lung cancer cases are expected to contribute 37% of male cancer cases in this region, while liver and lung will make up 34% and 29% of male cancer cases, respectively (Figure 5 and Figure 7; Table 2).

### 2.3. Age-Specific Incidence Trends in an Aging Population

**Figure 8:**
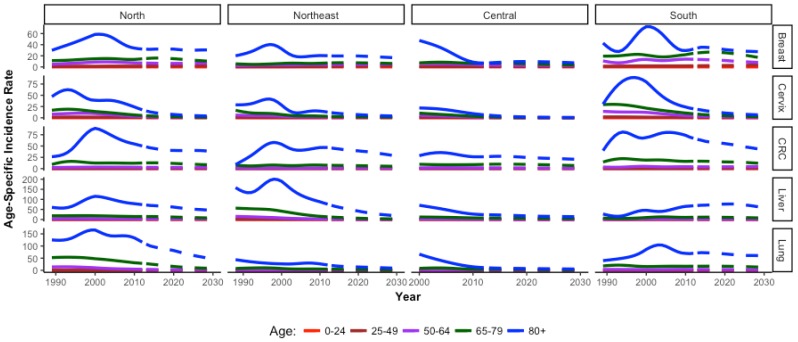
Age-specific incidence rates for each cancer in females only by region. Note the *Y*-axis are different for each cancer.

**Figure 9:**
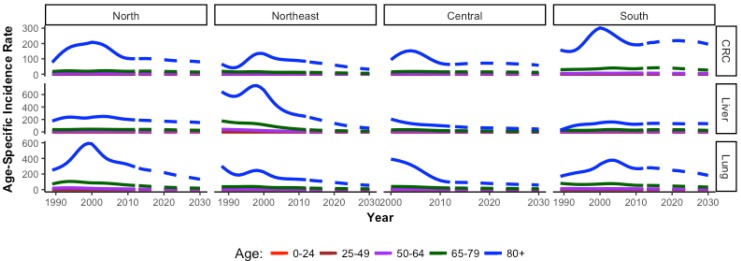
Age-specific incidence rates of each cancer in males only by region. Note the *Y*-axis are different for each cancer.

The overall summary figures mask interesting age-specific trends (Figure 8 and Figure 9). As expected, older age groups have higher incidence of cancer across regions compared to younger age groups. However, for males and females who are 80+ years, there is a peak in incidence at or around the year 2000 for almost all cancers in all regions. The central region has data available starting in 2000, however the decreasing trend at the start of the observed period indicates a potential peak prior to data collection. In females, a peak is evident in the oldest group for all cancer types in all regions except for lung cancer in the northeast and cervical cancer in the central region, which show stable incidence trends. In males, a peak for the oldest age group is evident in all regions for all cancer types, except liver cancer. Liver cancer incidence in older males exhibits a peak in the northeast region only. In younger age groups, peaks are evident for certain age groups and certain cancers, but lack an overall trend (Figures S1 and S2).

## 3. Discussion

National estimates for Thailand for these top five cancers have been previously reported, however they are limited to single estimates of each cancer over a few years at a time [23,24,25] and are reported only for specific registries [3,4,5,6,7,26,27,28], making comparisons by regions across cancer types difficult. This paper provides a comprehensive overview of national and subnational trends of top cancers in Thailand, including information on past and future temporal trends.

Although national estimates begin in 2000, regions with data in earlier years show increasing incidence of the five cancers combined. Rising incidences of cancer are partially attributable to large improvements of the healthcare system over several decades. In 1975, free health care was provided for the poor, but this policy was ineffective due to a lack of infrastructure. The 5th National Economic and Social Development plan (1982–1986) established infrastructure by freezing investment in urban hospitals, focusing funds on building rural district hospitals and community health centers, and employing and training doctors and community health workers at the village level [29,30]. At the end of this period, 92% of all districts had hospitals, 98% of subdistricts contained health centers and 91% of villages had trained health village volunteers [31]. The 6th (1987–1991), 7th (1992–1996) and 8th (1998–2001) plans, focused on improving equity of service delivery, health financing through insurance schemes, and focusing on preventive and primary care [31,32,33]. This infrastructure development culminated in effective implementation of the UHC system, which comprised of several insurance schemes and covered 97% of the population in 2002 [13]. Financial protection combined with improved access and equity of healthcare services combined on the local, provincial and national levels played a substantial role in detection of cancers.

With improved detection, the cancer trends shown here offer considerable information on the magnitude and change of incidence over time. It is crucial to quantify these measures so as to provide evidence from high-quality data that can be used to address the growing cancer burden. This work also provides a context within to understand risk factor profiles subnationally. We showed that breast, cervical and colorectal cancers have similar incidence across regions, whereas liver and lung cancers have significant burdens in particular regions, suggesting that risk factor profiles for some cancers are changing on a national level, while others are more region-specific. The changing lifestyles, behaviors and population structure of Thailand have implications specific to each cancer.

Breast cancer incidence increased significantly in all regions, ranging from 3% to 7% increases per year, depending on region. This is likely due to the general shift in reproductive risk factors in Thailand and variations by region. The total fertility rate (TFR) nationally Thailand was 1.8 in 2011, and 1.7, 1.8, 2.0 and 2.2 for the north, central, south and northeastern regions, respectively [11]. In the south, Muslim women had consistently higher TFRs compared to their Buddhist counterparts [34,35]. Overall, the national TFR decreased from 6.1 in 1960 to 1.6 in 2016 [11,36,37]. Age at menarche decreased from 12.4 years in 1997 to 11.8 years in 2017 [38,39]. The proportion of women who exclusively breastfed for the six months is highest in the north (19.6%) and lowest in the central region (7.9%), while the proportion of women who continued to breastfeed for 1 and 2 years is highest in the south and lowest in the northern region [11]. In addition, 44.9% of women in Thailand are classified as overweight or obese, which are known risk factors of postmenopausal breast cancer [40,41]. In particular, the proportion of overweight and obese women is highest in the central region and lowest in the northeastern region [11]. Since approximately year 2002, breast cancer screening campaigns were promoted, mostly in the form of educational awareness and knowledge [42]. The government promoted guidelines regarding self-breast examinations (BSE) and clinical breast exams (CBE). Although these guidelines are not covered under the UHC system, adherence to BSE increased from 40.1% in 2007 to 67.6% in 2015 [43,44]. Increased screening could partially explain the increasing trend in incidence nationally, as more cases are captured. However, available resources and infrastructure limit uptake of opportunistic mammography according to screening guidelines and limit the extent screening affects incidence for this cancer. There are approximately 2823 CT, MRI, and mammography machines in Thailand, with almost a quarter of machines in Bangkok and the remaining spread throughout 76 provinces. Approximately 35% of these machines are in the private sector and not available to 75% of the population covered under the UHC scheme [13,45]. Irrespective of opportunity however, benefits of mammographic screening in LMICs are likely to be low [46]. Considering the expected increase in incidence from this cancer shown by our findings, improvements in adherence to BSE and, particularly, CBE can aid in early detection to reduce morbidity and subsequently, mortality, from this disease [46].

Significant decreases in national cervical cancer incidence are attributed to the establishment of the national cervical cancer screening program in 2002. This program was implemented in response to statistics showing that cervical cancer was the top cancer in women prior to 2002 [47,48]. This was likely due to increasing prevalence of human papilloma virus (HPV), the strongest risk factor for cervical cancer [49]. Nationally, HPV prevalence is 7%, but prevalence varies by region [50]. Prevalence of HPV by population is 6% in the north, 8.7% in Bangkok, 14.1% in the northeast and 3% in the south [51,52,53,54]. Although HPV16 and HPV18 are the most common types, HPV52 and HPV58 also have high prevalence in the Thai population [51,54,55,56,57]. Information over time is limited on other risk factors for cervical cancer, however reports indicate that age at first intercourse has dropped sharply in the past two decades. The proportion of men and women who had their first sexual intercourse before 15 years increased significantly for younger cohorts [58,59]. To address the high rates of cervical cancer, the national screening program was implemented to cover screening once every five years for women ages 30–60 years with a goal of reaching 80% population coverage [60]. Variations in the extent and initial decrease in cervical cancer incidence by region are likely due to opportunistic screening efforts by hospitals and health centers in some regions [61,62]. However, with the standardized screening provided by the national program, our findings show cervical cancer incidence decreased significantly in all regions, illustrating an overall effectiveness of the program. This finding corresponds with recent literature. Although there is no official evaluation protocol for the national screening program, a 67.4% coverage rate in 2010 was found by the Ministry of Public Health from national survey data [63]. Despite the variation of coverage within each region, our projections indicate that cervical cancer incidence will continue to decrease nationally [7,64,65]. However, our findings indicate that incidence rates in the northeast will stabilize in the future. This is likely an artifact of our analysis. This region is unique in that researchers from Khon Kaen University began a large-scale Thai cohort study in 1990, enrolling northeastern residents until 2001 and proactively offered cervical cancer screening as part of their data collection [66]. At the time, this was the only organized screening effort in the nation. The effects of this effort can be seen in the reduction of cervical cancer incidence early on in this region whereas the increase observed from 2004 to 2008 is likely due to the establishment of the national screening program and its increased coverage. The following decreases in incidence from 2008 to 2012 are the expected effects of a screening program. Our analyses method bases future cervical cancer projections on the last ten years of observed data due to the national screening program. Due to the trend in the last ten years in the northeast, projection rates appear to stabilize. In fact, it is likely that rates will continue to decrease in this region, as in all other regions, with continued implementation of the national screening program.

Colorectal cancer is increasing in both men and women nationally. Although rates have been lower than other cancers in this study, incidence significantly increased each year in all regions. It is difficult to assess the epidemiology of this increasing trend, as there are likely several contributing factors. Food patterns, such as acquisition and consumption patterns have changed in recent years. The Thai diet shifted from high consumption of fruits, vegetables, legumes and grains, to one with higher consumption of animal products, fats and sugar [67]. It is important to note, however, that Thailand’s rapid socio-economic development resulted in inequitable income distribution. Consumption of purchased and commercially influenced foods is likely higher in wealthier families and our trends may reflect a disease affecting this subset of the population. Increasing prevalence of overweight and obese individuals, increased prevalence of smoking and alcohol consumption also likely play a role in the increasing trends shown here [68,69,70,71]. Other factors may contribute to underdiagnosis of colorectal cancer such as misclassification of disease due to overlapping symptomology or competing causes of mortality. For example, while mortality from acute diarrhea has declined considerably due to improved healthcare access and coverage and oral rehydration therapy, incidence of acute diarrhea in adults has increased to 2024 cases per 100,000 PY in 2009 and accounted for 29% of the digestive diseases in 2010 [72,73]. Also, prevalence of irritable bowel syndrome (IBS) is 4.8% in the Thai population [10]. It is possible that diagnoses of colorectal cancers are missed due to diagnosis or treatment of these or other fatal infectious bowel diseases [74]. These factors highlight the importance of screening for this disease. Currently, there is no established national colorectal cancer screening program, however, there is clear recognition of this problem as several screening studies have been conducted to assess uptake and efficacy of screening methods in subjects aged 50 years and above [75,76,77]. From pilot testing of immunochemical fecal occult blood testing and follow-up colonoscopy, the detection rate of colorectal cancer was 3.7% [76]. Considering the increasing projected trends from this study and Thailand’s aging population (Figure 2), it is reasonable to conclude that this disease will play a large role in Thailand’s cancer burden in the future if no standardized population-wide screening methods are introduced.

Lung cancer continues to have significantly higher incidence in northern Thai men and women compared to all other regions. Although rates are projected to decrease, they are expected to remain higher in the north than all other regions. Tobacco consumption is a well known risk factor for lung cancer. From 1991 to 2007 the north and northeastern regions had the highest daily smoking prevalence of 34.2% and 34.8%, respectively [78]. Thailand began a strong national anti-smoking campaign in 1991 which has evolved to include an extensive set of control measures [12]. As a result, the proportion of cigarette smokers in Thailand decreased from 30.1% in 1976 to 23.7% in 2009, of which only 3.1% were females [45,79]. In the north, the daily smoking prevalence dropped to 18.4% in 2009 [80]. Although it takes decades to go from an exposure to a disease outcome, it is possible that the decreasing daily smoking prevalence in the north plays a role in the decreasing incidence we show here. Former smokers have decreased risk of lung cancer, specifically squamous cell carcinoma, compared to current smokers, although they don’t reach levels of never smokers [81]. Nationally, the proportion of female smokers has consistently been significantly lower than male smokers from 1976 to 2009, ranging from 2.0% to 6.1% in females compared to 38.8% to 55.3% of males [45]. Nevertheless, the highest smoking rates among females were consistently found in the north. In 1991, the prevalence of northern female smokers was 12.5% and dropped to 5.8% in 2009. Conversely, the lowest smoking rates among females in 2009 were found in the south and northeast regions, with prevalences of 1.0% and 2.9%, respectively [80]. Due to smoking increases in males, however, the south and northeast had the highest daily smoking prevalences (25.7%, 21.0%, respectively) compared to other regions. This corresponds to projections of increased incidence in lung cancer in southern males, although this was not seen in the northeastern males. Our findings show that lung cancer incidence has increased significantly for females in the south and northeast despite the low smoking rates in females in these regions. Although lung cancer is generally associated with smoking, this association is stronger for certain histological types, such as squamous cell carcinomas (SCCs). In never smokers, adenocarcinomas are the most common form of lung cancer [82,83,84,85]. Global cancer statistics estimate approximately 25% of lung cancers are not attributable to smoking and more than half of these cases are found in women [86]. Worldwide, there has been a decline in SCCs and an increase in adenocarcinomas [87]. Our findings here are consistent with global trends and suggest the increase in lung cancer in nonsmoking women may be due to a rise in adenocarcinomas. For example, a study in southern Thailand showed increasing trends in incidence of lung adenocarcinoma in both males and females and that the estimated absolute incidence rate of lung adenocarcinoma in males is higher than that of SCC, similar to other countries such as the US, Canada, Japan and Taiwan [26,88,89,90,91,92]. Risk factors also potentially differ. While changes in cigarette design might be attributed to increased lung adenocarcinomas in smokers, factors such as indoor air pollution due to coal-burning stoves, fuels such as wood or charcoal, type of cooking oil used during frying or deep frying have been implicated as risk factors for lung adenocarcinomas in nonsmoking Asian populations [93,94,95,96]. Future studies should assess histological subtype to verify these findings, as they may impact future policy measures for lung cancer prevention strategies.

Liver cancer in Thailand has long been recognized as a significant burden. In 1991, liver cancer was the top cancer in males and the third most common cancer in females. Our findings here show the highest incidence of liver cancer during that time was also in the northeast. Liver cancer is comprised of two main histological subtypes: hepatocellular carcinoma (HCC) and cholangiocarcinoma (CCA). Although we do not have histology information available for this study, the regional liver cancer trends presented corroborate histology-based literature. The etiology of these diseases is important to understand these trends. Hepatitis B (HBV) is the most frequent underlying cause of HCC. Hepatitis C (HCV) is also a major risk factor, however global prevalence of this virus is much lower than HBV [97]. People immune to HBV have substantially lower risk of HCC. Therefore, HBV vaccination is widely recognized as the most effective measure to prevent HBV infection [97,98]. In 1992, a national hepatitis B vaccination program was implemented. Thai infants were vaccinated at 2 and 6 months of age [99]. The prevalence rates of HBV decreased significantly, from 7.8% in 1988 to 2.6% in 2009 [100]. Our findings show national incidence trends of liver cancer started decreasing for males and females around 2004, potentially due to effects from the HBV vaccination program. A previous study shows that while similar incidence was found in all regions for hepatocellular carcinoma (HCCs), there was clear geographic variation of cholangiocarcinoma (CCA), with the highest rates in the northeast [101]. Our findings clearly corroborate this trend, as the incidence of liver cancer is the highest in the northeast region. The etiology of CCA in Asian countries is primarily linked to infections [102]. In Thailand, infection with *Opisthorchis viverrini* (OV)*,* a liver fluke, causes CCA. This parasite is found in raw and improperly fermented cyprinoid river fish, a staple of the diet in the northeast region. The prevalence of OV in 1981 was 34.6% in the northeast region, the highest of all regions in Thailand. This prompted a liver fluke control operation in 1987 designed to diagnose and treat infections [103,104]. In 1996, the prevalence in the north surpassed that of the northeast and by 2001, prevalence by region was 19.3%, 15.7%, 3.8% and 0% in the north, northeast, central and south regions, respectively [104,105]. Here we show that although incidence is decreasing, the northeast still has the highest rates of liver cancer, particularly in men, as expected for CCA [106]. Cancer takes decades to develop and it is understandable that the high past exposures contributed to incidence trends shown here. As OV exposure decreased due to targeted intervention programs in the northeast in the 1990s, we see a corresponding decrease in incidence decades later. Therefore, it is unsurprising that due to increased OV prevalence in the north over time, we are now seeing increases in liver cancer in men and women. The liver fluke is currently most prevalent in the north [105] and our projections highlight this by showing increasing liver cancer incidence in the future. Unexpectedly, we also note increasing liver cancer incidence in the southern region. Yeesoonsang et al. recently found CCA incidence increasing in females in southern Thailand and an overall higher proportion of CCA in females compared to males [27]. In addition, incidence of CCA increased and HCC decreased in males until recent years in the south when rates stabilized [27]. Our findings corroborate this study as we found female liver cancer incidence continuously increasing in the south, while male liver cancer increased until recent years, when it stabilized. Considering that the south has no prevalence of liver fluke, these findings highlight a CCA with epidemiology distinct from the liver fluke and should be assessed for adequate prevention.

Age-specific incidence trends highlight a time frame around the year 2000 when incidence peaked for elderly males and females in all regions across cancer types. This is likely due to the convergence of several factors targeted towards improving quality of life in the elderly. In 1992, the government developed policies targeted to the elderly [107,108]. In particular, the Ministry of Public Health Regulations instituted a policy of free health care for elderly people above age 60 [14,109]. These policies influenced the Eighth National Economic and Social Development Plan (1997–2001), the Declaration on older persons in Thailand in 1991, the 2nd National Plan for Older Persons (2002–2021) and The Act on Older Persons in 2003. These included policies on welfare benefits, improvements in healthcare and quality of life, economical well-being, social and fiscal aspects for the elderly [6,110,111]. In addition, infrastructure was improved. From 1995 to 1999, the number of hospitals and other medical establishments with beds community health centers increased by 36%, improving access in rural areas [112]. Improved coverage, transportation and infrastructure contributed to increased access to healthcare for the elderly. The peaks in incidence in the elderly around 2000 may indicate diagnosis of prevalent cancer cases as access improved. These findings highlight notable effects of these focused health policies on the aging Thai population and illustrate their utility in diagnosing a NCD that requires long-term health management. Cancer detection in elderly population contributes to improved quality of life and self-efficacy, particularly if cases are detected at early stages.

From our projections, we show that national incidences of all cancers except cervical cancer are expected to increase. Specifically, the northeast and central regions are expected to contribute the highest proportion of cancer cases for males and females to the national burden in 2025. Despite lower incidence rates, these regions are expected to have a larger number of cases compared to other regions. This point highlights that, aside from changes in risk factor distribution of each cancer, the influence of the changing population structure must be considered. Cancer risk increases with age and the proportion of elderly in the Thai population is expected to increase nationwide by 2025 (Figure 2). The shifts in proportion of population by age group are apparent nationally and within each region. Subnational variations in population structure contribute to cancer trends shown in this paper. The north is expected to have the highest incidence rates for all cancers combined for males and females, yet the central and northeast regions have the largest population sizes and thus, are expected to have the most number of cases. The central region has the largest proportion of younger, working age people, whereas the north and northeast have lower proportions of people in the 20–35 year age groups. This is presumably due to job seeking movement of these age groups. Determining these types of population dynamics has important implications for planning of the scope and magnitude of prevention strategies. 

There are several limitations in this study. The central region has the lowest representation due to the inclusion of the Lopburi cancer registry located outside of Bangkok. The decision to assess the central region incidence burden without the Bangkok registry was a careful one. Bangkok can be considered a separate entity from the established regions due to strong disparities in wealth, education and health resources. It is also a large metropolitan area, making cancer registry data difficult to assess. There are a large number of immigrants that do not receive a Thai ID and by default, are not included in the registry as well as high population transition between public and private hospitals. These factors make data quality difficult to assess. Although Lopburi provides a smaller representation, it provides more reliable estimates of the incidence burden in the central region. In addition, there are inherent limitations in projected estimates. Our projections are based on observed data; it is possible that trends may diverge in the future. However, these projections are useful for health economists and policymakers as they provide a baseline on which future potential policies addressing the cancer burden can be assessed.

This is the first comprehensive overview of national and subnational incidence trends of top cancers in Thailand and serves to provide critical information on two fronts. (1) While the estimates provide information for measuring the current and future trajectory of the cancer burden, is it important to understand how the effects of health policy, particularly universal health care and screening programs, contributed to these trends. Understanding the data presented here within the context of health policy allows us to determine effective strategies and provide evidence for future targeted approaches. (2) It is well known that the cancer burden will increase in Thailand and that this is due to changes in population risk factor profiles. Tracking the burden over time, particularly by subregion where risk factors diverge, provides a basis for understanding geographical variations and health disparities.

## 4. Materials and Methods

### 4.1. Cancer Registries

Cancer registry data was compiled through the Thai Cancer Information Network from 6 provincial cancer registries: Chiang Mai, Lampang, Khon Kaen, Lopburi, Surat Thani, and Songkhla. Chiang Mai is the second largest province in Thailand and is located in the northern region. This registry was established in 1986 and actively collects case information from 39 sources. Lampang is also located in the northern region. This registry was established in 1995 and utilizes active and passive case collection from 18 sources, covering the population of all 13 districts. Khon Kaen is located in the northeast region of Thailand. Its registry was established in 1988 and utilizes active and passive data collection methods from 28 sources covering all 26 districts. Lopburi is in the central region of Thailand. This registry was established in 2000 and uses active data collection from 13 sources for case compilation across all 11 districts. Surat Thani is the largest of the southern provinces. This registry was established in 2001 and uses active and passive data collection from 20 sources for cancer cases across all 19 districts. Songkhla is located on the Malay peninsula of the southern region of Thailand. This registry was established in 1989 and uses active data collection from 23 sources. Contrary to all other provinces mentioned which are predominantly Buddhist, the population in this province is 30% Muslim.

**Figure 10:**
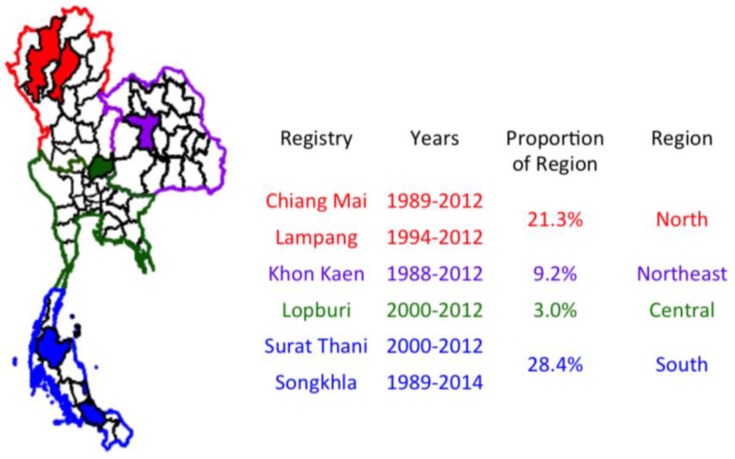
The Thai Cancer Information Network houses data from six high-quality cancer registries that were used to estimate subnational burdens of cancer as shown.

Chiang Mai and Lampang registries were combined to represent the northern region from 1989 to 2012, the Khon Kaen registry was used to represent the northeastern region from 1988 to 2012, the Lopburi registry represents the central region from 2000 to 2012 and Surat Thani and Songkhla registries were combined to represent the southern region from 1989 to 2014. Proportion of coverage of the population within each region are 22%, 9.2%, 3% and 28% for the north, northeast, central and south, respectively (Figure 10). The low representation of the central region is largely attributed to the process of registration and high population mobility of the Bangkok metropolitan area population. Non-national cases are not registered in the cancer registry, leading to misestimation of cancer trends and impractical projections of cancer trends in Bangkok. Lopburi is far enough away from Bangkok to avoid this and is the only registry in region with high data quality. Although Lopburi represents a smaller proportion of the central region, representation is more accurate of the population and thus, this registry was used to represent the central region.

### 4.2. Data Quality and Collection

Data from all registries was collected and assessed based on guidelines from the International Agency for Research on Cancer and the International Association of Cancer Registries, adapted to a middle-income context. Data was validated through clinical records, coded, and verified for duplication and multiple primaries according to guidelines. Cases were collected using ICD-10 codes for cancers of the liver (C22, C24), lung (C33–34), colorectal (C18, C19–20), breast (C50) and cervix (C53). The study was conducted in accordance with the Declaration of Helsinki, and the protocol was approved by the Thai Ethics Committee *(REC 58-013-18-1, 02/15/2016).*

### 4.3. Population

Population numbers of males and females for observed years were estimated from the two most recent population censuses surveyed by the National Statistical Office [113,114]. Intercensus populations were estimated using a log-linear function between two consecutive censuses. Projected population data was provided by the Office of National Economic and Social Development Board, which projected changes in the Thai population from 2010 to year 2030 using the data from the Population and Housing Census 2010, adjusting for fertility, mortality and migration. Population data was obtained for each registry and for each of the four regions of Thailand. 

### 4.4. Statistical Analysis

Age-specific incidence rates from representative registries were applied to the age-specific population of their respective region to obtain number of cases for each age group for each individual cancer type. Regional age-adjusted incidence rates for each cancer by region and sex were calculated from regional case tables and population and standardized to the world population proposed by Segi (1960), and later modified by Doll (1976) [115,116]. National age-adjusted incidence rates for each cancer by sex were calculated from summed cases and populations for each 5-year age group across regions and standardized to the modified Segi world population. National data was calculated from regional data, therefore, national incidence estimates are available only for the time in which data was available from all regions, 2000–2012.

Temporal trends in incidence for each region were identified by fitting joinpoint models to the log-transformed age standardized incidence rate (ASR) per 100,000 population [117,118]. Models were restricted to a maximum number of joinpoints (regional models: four joinpoints; Thailand models: two joinpoints) based on the number of years of data, to reduce the possibility of reporting artificial changes in trends. Trends were described in terms of annual percent change (APC). To assess whether the APC was statistically different from zero, a *t*-test based on asymptotic normality was used. Trends were described with terms such as “increasing” or “decreasing” when *p*-values of the slope for APC was statistically significantly at α = 0.05; if not, trends were described as “stable”. Average annual percent changes (AAPCs) were determined to provide magnitude and direction of change for years 2000–2012. This metric provides a summary measure of the annual percentage changes by weighting according to the length of segments in the model [119].

Age-period-cohort (APC) models were used to model and extrapolate cancer incidence rates to year 2030 as previously described [6,120,121]. Projected ASRs for breast, colorectal, liver and lung cancers were based off all observed data while projected ASRs for cervical cancer were based off of observed data from the most recent 10 years due to implementation of the national screening program in 2004. Observed data trends include both linear and residual trends. Linear trends represent drift, or the consistent average annual change in rates over time. The drift component is attenuated into the future to account for the idea that past trends will not continue indefinitely and has been shown to be a practical method of making future predictions [121]. The linear drift component of observed trends was attenuated with geometric dampening as described in Mistry 2011, reducing drift by 21.6%, 48.3% and 65.9% for each projected 5-year period, respectively [122]. The functions f_A_, f_P_ and f_C_ are natural cubic splines on 5-year intervals, which are used to smooth data for realistic interpolation at 1-year intervals. This approach was used for each cancer by region and sex. All ASRs are standardized to the modified Segi world population [115,116].

### 4.5. Standardized Rate Ratios

Standardized rate ratios (SRRs) and 95% confidence intervals (CI) were calculated based on Boyle’s calculations. [123]. SRRs were calculated from 2000 to 2012 as this was the time period where all registries had available data. 

Standardized Rate Ratios:

(1) $$SRR=\frac{ASR_{Regional}}{ASR_{Thailand}}$$

and 95% confidence intervals (CI) calculated by:

(2) $$95\%CI=SRR^{1\pm \left\{\frac{1.96}{\left(\frac{\left(ASR_{Regional}-ASR_{Thailand}\right)}{\sqrt{se{\left(ASR_{Regional}\right)}^{2}+se{\left(ASR_{Thailand}\right)}^{2}}}\right)}\right\}}$$

To estimate the number of cases occurring in each region and nationally in 2030, the projected age-specific incidence rates for each 5-year age group were multiplied by their respective population in the year 2030 for each cancer and summed. 

Terminology: “All cancers” and “combined cancers” refers only to cancers considered in these analyses: breast, cervix, colorectal, liver and lung. R-statistical software was used for analysis and projections (CRStat 3.2.0.1, epitools 0.5–7 and nordpred, R version 3.3.0) [124]. Joinpoint Regression software was used for trend analysis (v. 4.3.1.0) [119].

## 5. Conclusions

In the presence of the changing epidemiology of cancer in Thailand, universal healthcare, population dynamics, and existing health policies, our findings show the following: breast and colorectal cancers will contribute substantially to the national cancer burden in the future, cervical cancer incidence will continue to decline due to the national screening program, lung cancers increased in females in the northeast and south despite low smoking rates, and liver cancers increased in the north, where there is presence of liver fluke, and the south which is absent of liver fluke. In addition, we find that universal healthcare provides access for the elderly population in Thailand, a population commonly.

Within a global context, the strong cancer registration and healthcare infrastructure of Thailand offers a unique perspective on the ability to predict trends. Issues such as coverage and case capture are largely attenuated through the use of population-based registries and universal healthcare. Currently, many countries in the Southeast Asian Nations (ASEAN) are now seeing rapid economic development, similar to what Thailand has experienced in the past few decades. This work can help in understanding the cancer situation in other Southeast Asian countries, which have similar economic situations but don’t have this infrastructure, and where it might be headed in the future.

## Figures and Tables

**Table 1:** Subnational and National Age-standardized Incidence Rates for Cancers and Estimated New Cases, 2025, by Region.

Females															
	Age-Standardized Incidence Rate (ASR) (Per 100,000 Person-Years)					Cases									
				Change 2000–2012	Change 2012–2025				Change 2000–2012	Regional Proportion in 2012	Change 2012–2025	Regional Proportion in 2025	National Proportion		
	2000	2012	2025	ΔASR	ΔASR	2000	2012	2025	ΔCases	% All Cases	ΔCases	% All Cases	2012	2025%	Δ
North															
All Sites	93.4	88.9	81.8	−5%	−8%	6104.0	8362.4	9470.4	37%	100%	13%	100%	25%	21%	−3%
Breast	21.8	27.9	30.3	28%	9%	1475.6	2535.2	3102.5	72%	30%	22%	33%			
Cervix	17.5	13.5	6.8	−23%	−50%	1883.0	1208.6	562.2	−36%	14%	−53%	6%			
CRC	8.4	11.9	13.6	42%	14%	535.9	1120.3	1819.0	109%	13%	62%	19%			
Liver	10.0	14.1	14.3	41%	1%	625.9	1366.0	1901.7	118%	16%	39%	20%			
Lung	25.4	21.4	15.5	−16%	−28%	1583.6	2132.3	2085.0	35%	25%	−2%	22%			
Northeast															
All Sites	71.1	67.9	67.3	−5%	−1%	7335.2	9711.8	12,393.8	32%	100%	28%	100%	29%	28%	−1%
Breast	14.5	24.0	34.7	66%	45%	1606.8	3260.1	5076.9	103%	34%	56%	41%			
Cervix	17.3	9.2	11.7	−47%	27%	1508.3	1251.0	1613.2	−17%	13%	29%	13%			
CRC	7.7	8.9	12.0	16%	35%	773.0	1306.7	2555.3	69%	13%	96%	21%			
Liver	28.2	17.7	8.5	−37%	−52%	2779.2	2704.4	1762.3	−3%	28%	−35%	14%			
Lung	6.7	8.0	7.4	19%	−8%	667.9	1189.6	1386.1	78%	12%	17%	11%			
Central															
All Sites	68.7	72.9	75.1	6%	3%	7677.8	12,977.2	19,569.4	69%	100%	51%	100%	38%	44%	6%
Breast	20.7	29.4	33.7	42%	15%	2443.7	5335.2	8568.7	118%	41%	61%	44%			
Cervix	21.5	13.4	6.8	−38%	−49%	2417.6	2428.6	1368.7	0%	19%	−44%	7%			
CRC	9.2	11.4	17.5	24%	54%	925.0	1879.5	4839.2	103%	14%	157%	25%			
Liver	9.5	10.2	9.6	7%	−6%	1030.1	1796.7	2725.1	74%	14%	52%	14%			
Lung	7.9	8.5	7.9	8%	−7%	861.4	1537.2	2067.7	78%	12%	35%	11%			
South															
All Sites	57.5	52.0	66.4	−10%	28%	2267.3	2955.6	5566.0	30%	100%	88%	100%	9%	13%	4%
Breast	19.8	23.6	35.0	19%	48%	806.7	1298.3	2407.9	61%	44%	85%	43%			
Cervix	21.7	9.5	5.9	−56%	−38%	845.5	531.6	390.2	−37%	18%	−27%	7%			
CRC	8.5	7.9	13.3	−7%	68%	325.7	458.0	1105.2	41%	15%	141%	20%			
Liver	3.3	3.8	7.2	15%	89%	132.3	233.6	677.6	77%	8%	190%	12%			
Lung	4.1	7.2	10.9	76%	51%	157.1	434.1	985.1	176%	15%	127%	18%			
Thailand															
All Sites	82.7	83.6	71.1	1%	−15%	23,384.4	34,007.2	44,061.1	45%	100%	30%	100%	100%	100%	1%
Breast	19.2	26.2	33.9	36%	29%	6332.8	12,428.8	18,286.8	96%	37%	47%	42%			
Cervix	21.2	14.4	6.8	−32%	−53%	6654.3	5419.9	3264.8	−19%	16%	−40%	7%			
CRC	8.4	10.0	12.9	19%	29%	2559.7	4764.6	9125.2	86%	14%	92%	21%			
Liver	12.8	11.5	9.1	−10%	−21%	4567.6	6100.7	6462.5	34%	18%	6%	15%			
Lung	11.0	11.3	9.7	3%	−14%	3270.0	5293.2	6921.8	62%	16%	31%	16%			
Males															
	Age-Standardized Incidence Rate (ASR) (Per 100,000 Person-Years)					Cases									
				Change 2000–2012	Change 2012–2025				Change 2000–2012	Regional Proportion in 2012	Change 2012–2025	Regional Proportion in 2025	National Proportion		
	2000	2012	2025	ΔASR	ΔASR	2000	2012	2025	ΔCases	% All Cases	ΔCases	% All Cases	2012	2025%	Δ
North															
All Sites	67.1	86.6	84.2	29%	−3%	3839.5	7393.3	9437.3	93%	100%	28%	100%	25%	23%	−2%
CRC	9.4	14.9	18.6	59%	25%	549.4	1262.7	2071.9	130%	17%	64%	22%			
Liver	23.3	35.3	42.8	52%	21%	1359.8	3004.8	4477.1	121%	41%	49%	47%			
Lung	34.4	31.2	24.7	−9%	−21%	1930.3	3125.8	2888.3	62%	42%	−8%	31%			
Northeast															
All Sites	103.3	86.9	59.5	−16%	−32%	8826.7	10,911.1	10,345.0	24%	100%	−5%	100%	37%	25%	−12%
CRC	8.3	12.9	13.2	55%	2%	721.8	1603.9	2152.7	122%	15%	34%	21%			
Liver	72.0	55.0	31.1	−24%	−43%	6188.2	6918.1	4948.9	12%	63%	−28%	48%			
Lung	23.7	19.1	18.5	−19%	−3%	1916.7	2389.1	3243.4	25%	22%	36%	31%			
Central															
All Sites	62.8	61.5	67.7	−2%	10%	5711.0	8909.0	15,798.5	56%	100%	77%	100%	30%	38%	8%
CRC	8.6	17.0	25.5	98%	50%	731.5	2465.3	5820.2	237%	28%	136%	37%			
Liver	28.9	27.9	28.2	−3%	1%	2726.1	4120.1	5927.2	51%	46%	44%	38%			
Lung	25.3	16.7	18.6	−34%	11%	2253.4	2323.6	4051.1	3%	26%	74%	26%			
South															
All Sites	34.7	44.8	62.2	29%	39%	1175.1	2185.3	4425.4	86%	100%	103%	100%	7%	11%	4%
CRC	10.9	12.9	17.7	18%	37%	366.7	611.2	1281.2	67%	28%	110%	29%			
Liver	8.5	13.6	21.5	60%	58%	297.1	677.9	1496.6	128%	31%	121%	34%			
Lung	15.3	18.3	23.2	20%	27%	511.3	896.2	1647.6	75%	41%	84%	37%			
Thailand															
All Sites	73.5	72.8	69.2	−1%	−5%	19,552.3	29,398.6	41,137.2	50%	100%	40%	100%	100%	100%	
CRC	9.1	14.1	20.8	55%	48%	2369.5	5943.1	12,060.4	151%	20%	103%	29%			
Liver	32.9	32.2	30.6	−2%	−5%	10,571.1	14,720.8	16,821.0	39%	50%	14%	41%			
Lung	24.5	22.6	20.4	−8%	−10%	6611.7	8734.7	12,255.8	32%	30%	40%	30%			

**Table 2:** National and subnational trends in cancer incidence rates.

		Trend 1		Trend 2		Trend 3		Trend 4	
	Site	Years	APC	Years	APC	Years	APC	Years	APC
**FEMALE**									
Thailand									
	All Sites	2000–2008	0.8%	2008–2012	−2.6%				
	Breast	2000–2012	3.1% *						
	Cervix	2000–2008	−1.2%	2008–2012	−10.5% *				
	CRC	2000–2012	3.3% *						
	Liver	2000–2002	15.1% *	2002–2005	−10.0%	2005–2012	−2.1% *		
	Lung	2000–2012	−0.6%						
North									
	All Sites	1989–1994	10.3% *	1994–2003	1.6%	2003–2012	−0.9%		
	Breast	1989–2003	6.9% *	2003–2012	0.6%				
	Cervix	1989–1995	9.95% *	1995–2008	−1.4%	2008–2012	−12.5% *		
	CRC	1989–1994	16.4% *	1994–2012	3.1% *				
	Liver	1989–2012	2.4% *						
	Lung	1989–1993	11.7% *	1993–2012	−1.3%*				
Northeast									
	All Sites	1988–2012	−0.3%						
	Breast	1988–1999	7.1% *	1999–2012	4.2% *				
	Cervix	1988–2012	−2.3% *						
	CRC	1988–2012	3.5% *						
	Liver	1988–2002	−0.5%	2002–2012	−7.4% *				
	Lung	1988–1998	5.9% *	1998–2004	−5.9% *	2004–2012	4.5% *		
Central									
	All Sites	2000–2007	4.6% *	2007–2012	−4.8% *				
	Breast	2000–2012	2.8% *						
	Cervix	2000–2006	3.3%	2006–2012	−10.2% *				
	CRC	2000–2012	4.8% *						
	Liver	2000–2012	−0.1%						
	Lung	2000–2012	−0.8%						
South									
	All Sites	1989–2014	2.1% *						
	Breast	1989–2014	3.9% *						
	Cervix	1989–2000	2.1%	2000–2012	−5.0% *				
	CRC	1989–2014	4.0% *						
	Liver	1989–2014	5.5% *						
	Lung	1989–2014	4.7% *						
**MALE**									
Thailand									
	All Sites	2000–2012	−0.8 *						
	CRC	2000–2012	4.1% *						
	Liver	2000–2002	11.3%	2002–2005	−7.2%	2005–2012	−1.2%		
	Lung	2000–2012	−1.3% *						
North									
	All Sites	1989–1994	14.6% *	1994–2012	1.0%				
	CRC	1989–1995	16.0% *	1995–2012	3.3% *				
	Liver	1989–2012	3.8% *						
	Lung	1989–1994	16.8% *	1994–2012	−1.3% *				
Northeast									
	All Sites	1988–2002	−0.01	2002–2012	−3.4 *				
	CRC	1988–2012	2.8% *						
	Liver	1988–2002	−0.5%	2002–2008	−8.4% *	2008–2012	7.0%		
	Lung	1988–2001	4.3% *	2001–2004	−10.1%	2004–2012	2.3%		
Central									
	All Sites	2000–2007	3.6% *	2007–2012	−6.3%				
	CRC	2000–2012	5.5% *						
	Liver	2000–2008	3.6% *	2008–2012	−7.5% *				
	Lung	2000–2012	−1.8% *						
South									
	All Sites	1989–1997	0.1%	1997–2005	7.9% *	2005–2012	−1.0%	2012–2014	13.2%
	CRC	1989–2014	4.2% *						
	Liver	1989–1997	−1.2%	1997–2005	10.6% *	2005–2014	−1.6%		
	Lung	1989–1999	−0.1%	1999–2005	8.5% *	2005–2012	−2.6	2012–2014	17.4%

## Supplementary Materials

The following are available online at http://www.mdpi.com/2072-6694/9/8/108/s1, Figure S1: Age-specific incidence rates for each cancer in females only by region. The 80+ age category was removed. Note the *Y*-axis are different for each cancer, Figure S2: Age-specific incidence rates for each cancer in males only by region. The 80+ age category was removed. Note the *Y*-axis are different for each cancer.
