# Supplementary material for: National and Subnational Population-Based Incidence of Cancer in Thailand: Assessing Cancers with the Highest Burdens
Source: Cancers (Basel). 2017 Aug 17;9(8):108. doi: 10.3390/cancers9080108 (PMC5575611; doi:10.3390/cancers9080108)
Supplement: Supplementary file 1 [file cancers-09-00108-s001.pdf]

Supplementary Materials: National and Subnational Population-Based Incidence of Cancer in Thailand: Assessing Cancers with the Highest Burdens

Shama Virani, Surichai Bilheem,Wasan Chansaard, Imjai Chitapanarux, Karnchana Daoprasert, Somsak Khuanchana, Atit Leklob, Donsuk Pongnikorn, Laura S. Rozek, Surattaya Siriarechakul, Krittika Suwanrungruang, Sukit Tassanasunthornwong, Patravoot Vatanasapt and Hutcha Sriplung

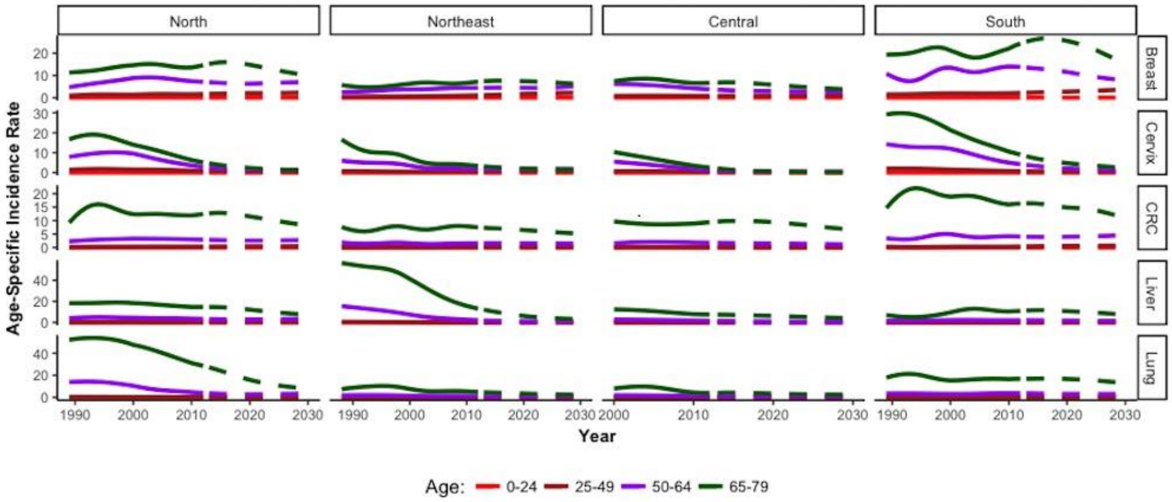

Figure S1. Age-specific incidence rates for each cancer in females only by region. The 80+ age category was removed. Note the Y-axis are different for each cancer.

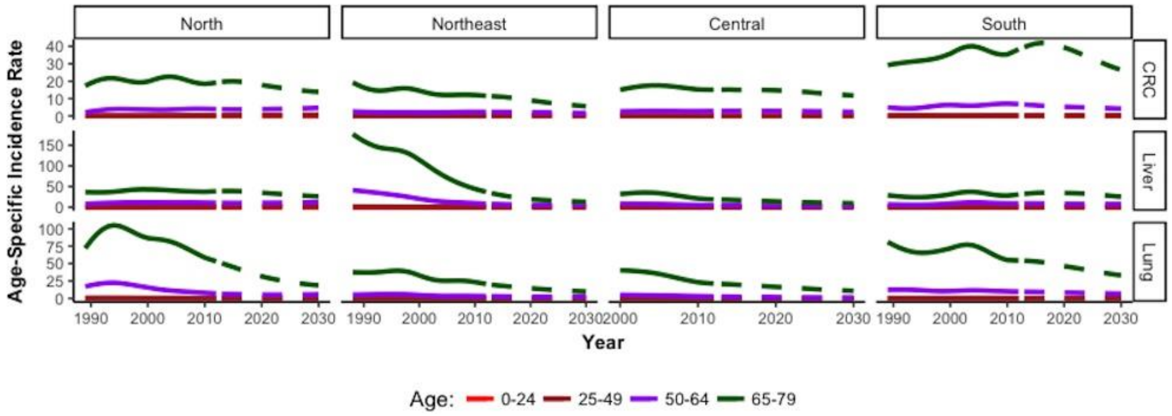

Figure S2. Age-specific incidence rates for each cancer in males only by region. The 80+ age category was removed. Note the Y-axis are different for each cancer.

© 2017 by the authors. Submitted for possible open access publication under the terms and conditions of the Creative Commons Attribution (CC-BY) license (http://creativecommons.org/licenses/by/4.0/).
